# Herbicide Resistance Genes in Crops: Mechanisms, Progress, and Future Perspectives

**DOI:** 10.3390/plants15172718

**Published:** 2026-09-04

**Authors:** Yongchao Guo, Xue Ma, Zihang Li, Chunhong Liu, Cheng Chu, Jinping Wang, Zhifang Wang, Zhiyin Jiao, Guoquan Liu, Peng Lv, Yannan Shi

**Affiliations:** 1 Institute of Millet Crops, Hebei Academy of Agriculture & Forestry Sciences, Hebei Branch of China National Sorghum Improvement Center, Shijiazhuang 050035, China; 2 China-Australia Joint Laboratory for Sorghum Transformation and Genome Editing, Shijiazhuang 050035, China; 3 Centre for Crop Science, Queensland Alliance for Agriculture and Food Innovation, The University of Queensland, St Lucia, Brisbane 4072, Australia

**Keywords:** weed classification, herbicides, resistance genes, herbicide resistance, genome editing, crop breeding

## Abstract

While previous reviews have largely focused on individual crops or single target-site mechanisms, the full-chain comparative landscape across major cereal crops remains unexplored. Here, we fill this critical gap by providing the first systematic, cross-crop comparative review that spans herbicide targets, resistance mechanisms, and breeding applications across four major cereals—rice, maize, wheat, and sorghum. Weed infestation is a serious constraint on crop production. Chemical weed control faces challenges such as herbicide resistance evolution and ecological risks. Developing herbicide-resistant varieties is a fundamental approach to achieve green and sustainable weed management. This review systematically summarizes research progress on herbicide resistance genes from three aspects: herbicide classification, resistance mechanisms, and crop breeding applications. It highlights key differences among four major cereal crops (rice, maize, wheat, and sorghum) in resistance-gene discovery and translational progress. Rice has the richest target-site resistance-gene resources. Maize leads in commercialization of transgenic herbicide resistance. Wheat focuses on endogenous precise editing due to genome complexity and regulatory constraints. Sorghum relies on specific mutations to serve cereal–legume intercropping systems. Based on this comparison, this review identifies the core trends in resistance breeding: from single-gene to multi-gene stacking, and from exogenous gene introduction to endogenous gene editing. It also points out common bottlenecks, including insufficient systematic mining of resistance-gene resources, lagging elucidation of non-target-site resistance regulatory networks, and strong genotype dependence in genetic transformation. Future efforts should focus on exploring broad-spectrum resistance genes, optimizing precise editing technologies, and developing sustainable resistance management strategies. This review provides a theoretical framework and practical references for molecular breeding of herbicide-resistant crops.

## 1. Introduction

Weeds are a major constraint on global agricultural productivity. According to the Food and Agriculture Organization (FAO), approximately 50% of the world’s land area is suitable for agricultural production. On this arable land, farmers face numerous challenges, including pests and weeds, with weeds being a critical factor constraining agricultural productivity [1]. Weeds in farmland compete with crops for essential resources including nutrients, space, light, and water. Depending on the plant growth stage and the duration of weed infestation, weeds can reduce crop yields by 20–50% [2,3]. Effective weed control is fundamental to global food security.

Currently, a range of weed management strategies are available for weed control, including physical, mechanical, biological, and chemical approaches. Physical weeding, including flame- and laser-based methods, enables efficient and precise weed removal without the use of chemical herbicides. However, flame weeding has the disadvantages of high carbon emissions and a high risk of causing fires, while the high-energy beam of laser weeding may cause accidental injury to humans and animals [4]. Traditional mechanical weeding is prone to causing crop damage [5]. Biological weeding mainly utilizes plant extracts and pathogens to produce bioherbicides, but it suffers from high production costs, and some formulations are toxic to mammals [6]. Considering control costs, the adaptability of mechanized equipment for field application, and the core production goals of rapid weed control, cost reduction, and efficiency improvement, chemical weeding remains the most widely adopted and effective weed management strategy globally at present [7,8]. Therefore, the application of chemical herbicides has become an important part of modern agricultural development, playing a crucial role in reducing labor costs and increasing crop yields [9,10].

However, long-term reliance on chemical herbicides also poses serious problems. Although herbicides play a vital role in enhancing crop productivity and safeguarding food security, their release into the environment poses a serious threat to human health and natural ecosystems [11]. According to the FAO, global agricultural pesticide use amounted to 3.73 million tons of active ingredients in 2023, a figure that represents a doubling since 1990 and a 14% increase over the past decade, with herbicides comprising approximately 51% of the total. Improper or excessive herbicide application promotes residue accumulation in soil and plants, thereby inducing crop phytotoxicity and accelerating the evolution of herbicide-resistant weed populations. By 2025, the International Herbicide-Resistant Weed Database had recorded 534–539 unique resistance cases, involving 273 weed species, 98–102 crops, and 72–75 countries globally [12,13]. Thus, balancing effective weed control with minimizing crop and environmental damage is a core challenge for herbicide development.

To address these challenges, several strategies have been proposed in recent years, including the development of novel herbicides, the breeding of herbicide-resistant crops, the enhancement of crop competitive ability, and the application of systemic and pre-emergence herbicides [14]. Systemic herbicides are absorbed and translocated throughout the plant for efficient control, whereas pre-emergence herbicides inhibit weed seed germination, reducing early competition and the need for subsequent applications [15,16]. The most environmentally sustainable and effective approach remains the development of herbicide-resistant varieties across different crops. The identification of herbicide resistance genes through molecular biology techniques, elucidation of their functions and mechanisms, and the breeding of resistant varieties constitute a major research direction for sustainable weed management. Accordingly, the primary objective of this review is to systematically synthesize recent research advances in herbicide resistance genes in major crops, covering their classification, molecular mechanisms, and progress in crop breeding. In addition, we summarize previous findings, analyze current bottlenecks and challenges, and discuss future research directions and application prospects. Through this comprehensive overview, we provide a theoretical reference for the breeding of herbicide-resistant crop varieties and the development of effective weed management systems, thereby serving as a bridge to the subsequent detailed discussion of resistance-gene research and breeding strategies.

## 2. Classification of Common Field Weeds

Field weeds pose a major threat to crop production by competing for water, nutrients, and light, while also serving as reservoirs for pests and diseases [17]. Based on morphological and taxonomic characteristics, they are broadly categorized into dicotyledonous (broadleaf) and monocotyledonous weeds. Dicotyledonous weeds, such as *Chenopodium album*, *Amaranthus retroflexus*, and *Xanthium strumarium*, are the most diverse and damaging group in agroecosystems [18,19]. Monocotyledonous weeds mainly comprise grasses (Poaceae) and sedges (Cyperaceae), with *Echinochloa crus-galli* and *Leptochloa chinensis* being the most dominant malignant weeds in paddy fields [20,21,22,23].

Notably, prolonged and repeated herbicide use has led to a significant increase in resistant biotypes among both dicotyledonous (e.g., *Descurainia sophia* and *Galium aparine*) and monocotyledonous species (e.g., *Eleusine indica* and *Cyperus difformis*), thereby raising the challenge of chemical control and driving the urgent need for resistance-gene discovery [24].

## 3. Classification of Herbicides

The preceding sections have described the major types of weeds and their impacts in crop fields. To manage different weeds effectively, a clear understanding of herbicide modes of action is essential. The internationally accepted classification system, established by the Herbicide Resistance Action Committee (HRAC), categorizes herbicides by their target sites (https://hracglobal.com/tools/2026-hrac-global-herbicide-moa-classification accessed on 18 May 2026). Following this framework and considering the active ingredients registered in China, we classify herbicides into two major groups: photosynthetic inhibitors and biosynthesis inhibitors. The following sections provide a systematic overview of the target sites and representative herbicides for each category.

### 3.1. Photosynthetic Inhibitors

Photosynthesis sustains plant productivity and plays a key role in terrestrial carbon and oxygen cycles [25]. Photosynthesis-inhibiting herbicides (Table 1), also known as photosystem inhibitors, function primarily by blocking the photosynthetic electron transport chain and inhibiting photophosphorylation, thereby preventing weeds from synthesizing organic compounds and leading to their death. These are commonly used non-selective herbicides in agricultural fields [26]. Based on their target sites, these herbicides are further divided into Photosystem II inhibitors, Photosystem I inhibitors, and pigment synthesis inhibitors [27,28].

#### 3.1.1. Photosystem II Inhibitors

Photosystem II (PSII) inhibitors primarily target the PSII reaction center in the thylakoid membrane of chloroplasts. Their mode of action involves herbicide molecules competing with the QB binding site on the D1 protein, altering its amino acid structure and blocking electron transfer to plastoquinone. This interruption of the photosynthetic electron transport chain prevents photophosphorylation [29]. These herbicides are further divided into two groups based on their binding sites. Those in the first group, Serine 264 binders (and other non-histidine 215 binders), interact with Serine 264 and non-histidine 215 residues of the D1 protein, and include triazines (ametryn, atrazine, prometryn), ureas (chlortoluron, diuron, isoproturon), triazinones (hexazinone, metamitron, metribuzin), uracils (bromacil), phenylcarbamates (desmedipham, phenmedipham), amides (propanil), and triazolones (amicarbazone). Those in the second group, Histidine 215 binders, interact with the Histidine 215 residue, and include nitriles (bromoxynil, ioxynil octanoate, bromoxynil octanoate) and benzothiadiazinones (bentazone) [30,31].

#### 3.1.2. Photosystem I Inhibitors

These herbicides target the photosystem I (PSI) reaction center in the thylakoid membrane of chloroplasts. Their mode of action involves blocking electron transfer from PSI to ferredoxin, thereby preventing the reduction of NADP^+^ to NADPH. As a result, high-energy electrons accumulate and react with oxygen to produce reactive oxygen species (ROS), such as superoxide anion and hydroxyl radicals. ROS are highly oxidative and damage the phospholipid bilayer of cell membranes, leading to increased membrane permeability. This causes substantial leakage of electrolytes and metabolites, resulting in cell necrosis and ultimately weed wilting and death [32]. Representative herbicides in this class include pyridinium compounds, such as diquat and diquat dichloride. These herbicides act rapidly and have a broad weed control spectrum, but they are highly toxic to humans and animals. Therefore, strict compliance with safety regulations and handling procedures is required during their application [33].

#### 3.1.3. Pigment Synthesis Inhibitors

These herbicides block chlorophyll or carotenoid biosynthesis in plants, causing loss of green color or bleaching due to pigment deficiency. As a result, affected plants are unable to photosynthesize and eventually die. They act primarily on the photosynthetic pigment synthesis system and fall into three categories: carotenoid synthesis inhibitors, chlorophyll synthesis inhibitors, and plastoquinone biosynthesis inhibitors. Among these, carotenoid synthesis inhibitors are the most widely used class [34].

Carotenoids are protective pigments in the plant photosynthetic system, preventing chlorophyll from being damaged by photooxidation [35]. These herbicides inhibit carotenoid biosynthesis, indirectly causing rapid chlorophyll degradation and resulting in complete plant bleaching. Their primary targets are phytoene dehydrogenase (PDS) and lycopene cyclase. PDS inhibitors can be divided into two types based on their mode of action: direct and indirect inhibitors. Direct PDS inhibitors include phenyl ethers (diflufenican, picolinafen), N-phenyl heterocycles (flurochloridone), and diphenyl heterocycles (fluridone, flurtamone). Indirect inhibitors act through four target sites: (1) deoxy-D-xylulose phosphate synthase inhibitors, such as the isoxazolidinone compounds clomazone and bixlozone; (2) 4-hydroxyphenylpyruvate dioxygenase inhibitors, including triketones (benzobicyclon, mesotrione, sulcotrione), pyrazolones (topramezone, cypyrafluone, fenpyrafluone), isoxazolones (isoxaflutole), and oxazoles (flusulfamide, pyrasulfotole); (3) solanesyl diphosphate synthase inhibitors (the isoxazole compound pyrimisulfan); and (4) homogentisate solanesyltransferase inhibitors [36].

Chlorophyll synthesis inhibitors act by directly blocking key steps in chlorophyll biosynthesis, preventing normal chlorophyll production and causing leaf chlorosis and yellowing [37]. Their target is protoporphyrinogen oxidase (PPO), a key enzyme in the chlorophyll synthesis pathway [38]. Common herbicides in this class include N-phenyl-phthalimides (flumioxazin, fluthiacet-methyl, pentoxazone), diphenyl ethers (acifluorfen, fomesafen, lactofen), N-phenyl-triazolinones (carfentrazone-ethyl, sulfentrazone), N-phenyl-oxadiazolones (oxadiazon, oxadiargyl), phenylpyrazoles (pyraflufen-ethyl), and pyrazolopyrazoles (pyraclonil).

### 3.2. Biosynthesis Inhibitors

Biosynthesis-inhibiting herbicides are agents that exert herbicidal effects by interfering with core biochemical metabolic pathways in plants (Table 2). They represent an important tool in modern agriculture for weed control and crop protection [39]. Based on their target sites and biochemical mechanisms, these herbicides are divided into amino acid synthesis inhibitors, fatty acid synthesis inhibitors, microtubule inhibitors, auxinic inhibitors, and other biosynthesis inhibitors.

#### 3.2.1. Amino Acid Synthesis Inhibitors

This category is one of the most widely used types of herbicides. Their core action is to block the biosynthetic pathways of essential amino acids in plants, causing weed death due to impaired protein synthesis and arrested cell division. Based on the target enzymes, these herbicides are mainly divided into three categories. The first category targets acetolactate synthase (ALS), also known as acetohydroxyacid synthase (AHAS). ALS is the key enzyme that catalyzes the first step in the biosynthesis of branched-chain amino acids (BCAAs), which include leucine, valine, and isoleucine. Inhibition of ALS activity disrupts BCAA synthesis and ultimately leads to plant death [40,41]. Major representatives in this group include imidazolinones (imazamox, imazapic, imazapyr), triazolopyrimidines Group 1 (cloransulam-methyl, diclosulam, florasulam), triazolopyrimidines Group 2 (penoxsulam, pyroxsulam), triazolinones (flucarbazone-sodium, thiencarbazone-methyl), sulfonylureas (pyrazosulfuron-ethyl, rimsulfuron, sulfometuron-methyl, tribenuron-methyl, flucetosulfuron), sulfonanilides (triafamone), and pyrimidinylbenzoates (bispyribac-sodium, pyriminobac-methyl, pyriftalid) [42,43]. Imidazolinones are systemic herbicides commonly used in crops such as soybean and peanut [44].

The second class comprises 5-enolpyruvylshikimate-3-phosphate synthase (EPSPS) inhibitors. EPSPS is a key rate-limiting enzyme in the shikimate biosynthesis pathway of plants, responsible for catalyzing the condensation of shikimate-3-phosphate and phosphoenolpyruvate to form EPSP, which is then involved in the synthesis of aromatic amino acids (phenylalanine, tyrosine, tryptophan) essential for plant growth and development [45]. This enzyme is the specific target of organophosphorus herbicides. Common examples include glyphosate, glyphosate ammonium, glyphosate dimethylamine salt, and glyphosate potassium salt.

The third category targets glutamine synthetase (GS). GS is a key detoxifying enzyme present in all plant tissues, which alleviates ammonium toxicity generated from amino acid degradation, photorespiration, and nitrate reduction [46]. Common herbicides in this class are phosphinic acids, mainly including glufosinate and glufosinate-P.

#### 3.2.2. Fatty Acid Synthesis Inhibitors

These herbicides act primarily by inhibiting key enzymes involved in plant fatty acid biosynthesis, thereby disrupting cell membrane and lipid synthesis, which leads to meristem necrosis and growth arrest in weeds [47]. They mainly include three categories. The first category targets acetyl-CoA carboxylase (ACCase), the rate-limiting enzyme in fatty acid synthesis in grasses [48]. ACCase catalyzes the conversion of acetyl-CoA to malonyl-CoA. These herbicides bind specifically to the carboxyltransferase subunit, inhibiting ACCase activity and blocking the synthesis of lipids and certain secondary metabolites, thereby serving as selective herbicides for grass weed control [49]. These include cyclohexanediones (clethodim, sethoxydim, tralkoxydim), aryloxyphenoxypropionates (pinoxaden, haloxyfop-methyl, fluazifop-P-butyl, fenoxaprop-P-ethyl, quizalofop-P-ethyl), phenylpyrazolines (pinoxaden), and bicyclooctanetriones (Metproxybicyclone).

The second type comprises very-long-chain fatty acid (VLCFA) elongase inhibitors. Their mechanism is to specifically inhibit the activity of 3-ketoacyl-CoA synthase (KCS) in the VLCFA elongase complex in the plant endoplasmic reticulum, covalently modifying and inactivating the cysteine residue at the active center of the condensing enzyme, thereby blocking the synthesis of VLCFAs with carbon chain lengths >18 [50,51]. These include α-chloroacetamides (alachlor, butachlor, metazachlor, metolachlor, pretilachlor), thiocarbamates (dimepiperate, molinate, thiobencarb, triallate), benzofurans (ethofumesate), isoxazolines (pyroxasulfone), α-oxyacetamides (mefenacet, flufenacet), α-thioacetamides (anilofos), and sulfonamides (dimethenamid). The third category targets fatty acyl-ACP thioesterases, inhibiting the release of fatty acids from acyl carrier proteins and thereby blocking normal fatty acid synthesis. Representative herbicides in this class include diphenyl heterocycles such as oxaziclomefone.

#### 3.2.3. Microtubule Inhibitors

These herbicides act primarily by disrupting microtubules, thereby affecting cell division and expansion during plant growth, ultimately leading to cell rupture and plant death [52,53]. They can be divided into two categories: microtubule assembly inhibitors and microtubule disruptors. Microtubule assembly inhibitors suppress microtubule assembly and spindle fiber formation, thereby arresting mitotic cell division. Common herbicides in this class include dinitroanilines (butralin, prodiamine, pendimethalin), benzamides (pronamide), and pyridines (dithiopyr, thiazopyr).

#### 3.2.4. Auxinic Inhibitors

Auxinic inhibitors are selective herbicides used primarily in monocot crops. They can be divided into two categories: auxin mimics and auxin transport inhibitors. Auxin mimics act similarly to endogenous auxins. They acidify the cell wall by stimulating membrane-bound ATPase proton pumps, inducing cell elongation, and ultimately causing abnormal growth and plant death [54]. Common auxin mimics include phenoxycarboxylic acids (2,4-D sodium salt, 2,4-D 2-ethylhexyl ester, MCPA dimethylamine salt), 6-chloropicolinic acids (aminopyralid, clopyralid, picloram), 6-arylpicolinic acids (florpyrauxifen, halauxifen), quinolinecarboxylic acids (quinclorac), benzoic acids (dicamba), and pyridyloxycarboxylic acids (fluroxypyr, triclopyr, fluroxypyr-meptyl). Auxin transport inhibitors, on the other hand, disrupt auxin homeostasis by inhibiting the polar transport of indole-3-acetic acid (IAA).

#### 3.2.5. Other Key Biosynthesis Inhibitors

In addition to the categories described above, some herbicides act by interfering with other core metabolic pathways in plants. These include cellulose biosynthesis inhibitors (CBIs), which target growing plant cells and suppress cellulose synthesis [55]. Dihydropteroate synthase inhibitors block normal folate synthesis, thereby affecting DNA and RNA synthesis. Dihydroorotate dehydrogenase inhibitors disrupt the oxidation of dihydroorotate to orotate, impairing pyrimidine synthesis and ultimately causing plant death.

The above classification by target sites provides a molecular framework for understanding where and how herbicides act. However, all herbicides face a common challenge in the field: the evolution of weed resistance. When herbicides continuously impose selection pressure on weed populations, individuals carrying resistant genotypes survive and reproduce. Depending on whether the genetic alteration occurs in the target protein itself or in other metabolic pathways, resistance mechanisms are divided into two fundamental categories: target-site resistance (TSR) and non-target-site resistance (NTSR). This classification directly guides the subsequent section on resistance-gene discovery and breeding applications in major crops.

## 4. Classification of Herbicide Resistance Genes

**Figure 1:**
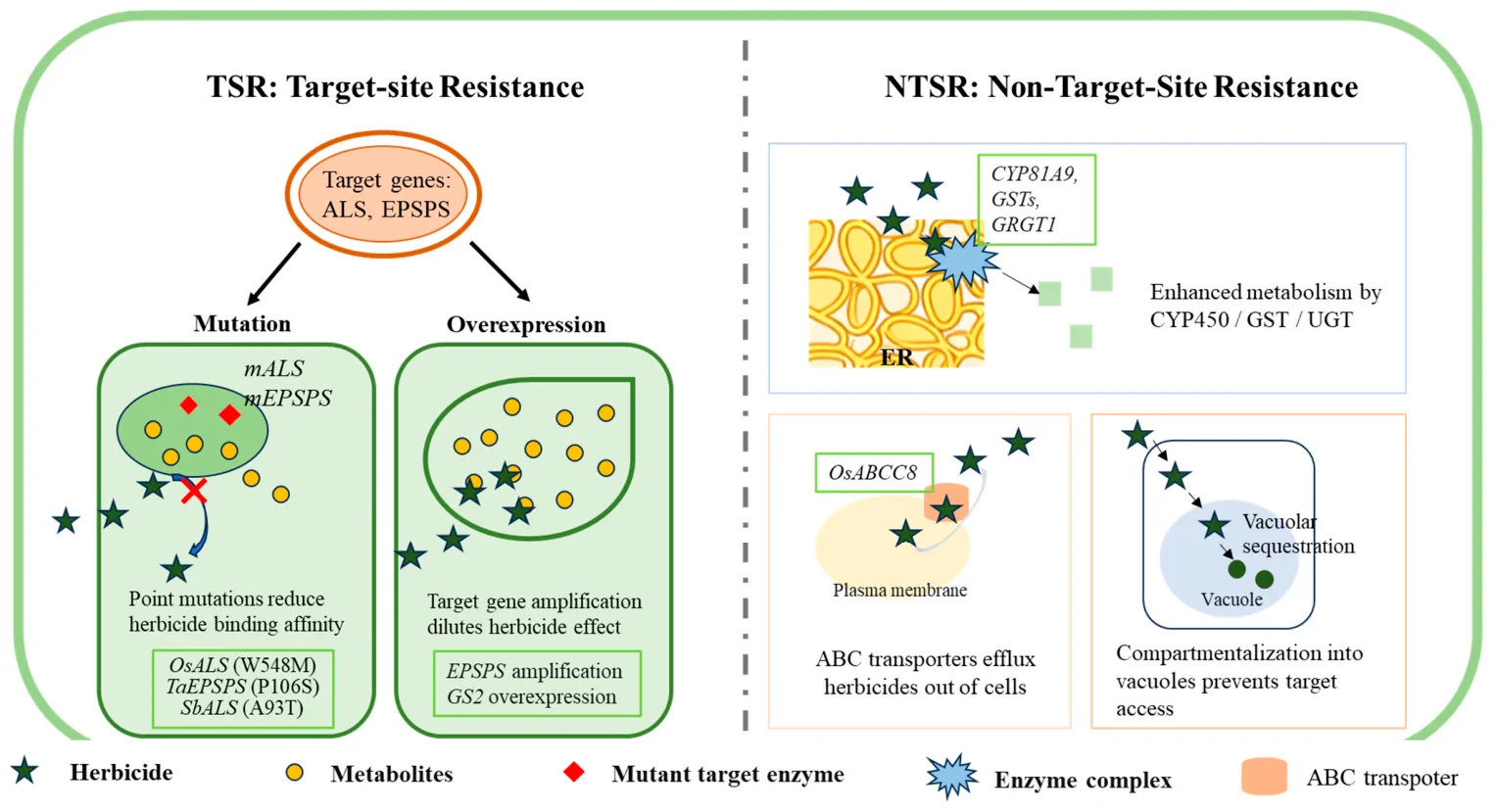
Molecular mechanisms of herbicide resistance in plants: target-site resistance (TSR) and non-target-site resistance (NTSR).

The preceding sections have classified herbicides by their target sites. However, all herbicides face a common challenge in the field: the evolution of weed resistance. Continuous use of herbicides with the same or similar modes of action imposes sustained selection pressure on weed populations. Individuals carrying resistant genotypes survive and reproduce, leading to the spread of resistant populations. With the widespread and prolonged use of herbicides, weed resistance has become increasingly prominent and is now a major threat to global food security. Weed resistance to herbicides is a genetic adaptive capacity evolved under long-term selection pressure. Based on the underlying mechanisms, it can be classified into two major categories: target-site resistance and non-target-site resistance (Figure 1) [56,57].

### 4.1. Target-Site Resistance

Target-site resistance (TSR) arises from structural or expression-level alterations in the herbicide’s target protein, leading to reduced or loss of herbicide binding to the target, thereby conferring resistance in weeds [58]. This type of resistance is primarily caused by mutations in the genes encoding the target protein or alterations in their expression levels. Based on the molecular mechanisms, TSR can be further divided into two categories. The first category involves mutations in genes encoding the target protein. To date, various types of target protein mutations have been found to be closely associated with herbicide resistance in weeds, the majority of which are single-gene mutations, such as those in ALS, ACCase, and EPSPS [42,45,49]. In studies on resistance to ALS-inhibiting herbicides, Pro197-Ser or Pro197-Thr substitution mutations in the *ALS* gene of Bromus populations directly conferred extremely high resistance levels to multiple ALS inhibitors [58]. In *Amaranthus hybridus* populations, a triple mutation in the *EPSPS* gene (Pro-106, Thr-102, and Ala-1033) allowed plants to survive under 32-fold concentrations of glyphosate [59]. The second category involves overexpression of the target gene. By enhancing the transcription and translation of the target gene, the abundance of the target protein is increased, thereby reducing the toxic effect of the herbicide on weeds. Target gene overexpression is also an important form of TSR. In glyphosate-resistant *Amaranthus palmeri*, EPSPS copy number was 5- to 160-fold higher in resistant individuals than in susceptible ones, and this overexpression conferred significant resistance to glyphosate [60]. In triploid Cyperus rotundus, resistance to glufosinate was demonstrated to result from overexpression of the target gene GS2, which dilutes the inhibitory effect of the herbicide by increasing the amount of target protein [61].

### 4.2. Non-Target-Site Resistance (NTSR)

Non-target-site resistance does not involve alterations in the herbicide target protein; instead, it reduces the effective concentration of herbicide reaching the target site through a series of physiological and biochemical processes, including enhanced herbicide metabolism, reduced uptake and translocation, and sequestration [62]. The first mechanism involves enhanced metabolic capacity. Resistant weeds upregulate the expression of metabolic enzyme genes to enhance their ability to degrade herbicides. Among these, cytochrome P450 enzymes, glutathione S transferases (GSTs), aldo keto reductases, and ABC transporters are core metabolism-related genes. In *Echinochloa crus-galli* populations resistant to cyhalofop-butyl, studies found that resistance was associated with significantly increased GST activity and accelerated metabolic rates, and multiple upregulated GSTs, cytochrome P450, and ABC transporter genes were identified [63]. The second mechanism involves reduced herbicide uptake and translocation. By altering the leaf surface structure or the activity of uptake carriers, weeds reduce the amount of herbicide absorbed, thereby minimizing the impact on physiological processes. In glyphosate-resistant *Amaranthus palmeri* populations, resistant plants retained most of the herbicide in the leaf tissues, whereas susceptible plants exhibited significantly higher herbicide uptake [64]. The third mechanism is herbicide sequestration. Herbicide or its metabolites are compartmentalized within specific cellular structures, such as vacuoles, preventing contact with the target protein and reducing toxicity [65]. In glyphosate-resistant *Echinochloa glabrescens* populations, an ABC transporter gene EcABCC8 was identified. This transporter is localized to the plasma membrane and effluxes glyphosate from the cytoplasm to the apoplast, thereby reducing intracellular glyphosate concentration [66].

## 5. Progress on Herbicide Resistance Genes in Major Crops

### 5.1. Progress on Herbicide Resistance Genes in Rice

Rice is a staple food crop worldwide. Weed infestation severely constrains its yield and quality, and the widespread use of herbicides has driven systematic research on herbicide resistance genes in rice. Current studies on rice herbicide resistance mainly focus on two core mechanisms: TSR and NTSR.

For TSR, multiple resistance genes have been identified in response to herbicides with different modes of action. For example, specific mutations in the ACCase gene confer resistance to ACCase-inhibiting herbicides such as cyhalofop-butyl and metamifop [67]. However, most ACCase resistance mutations identified in rice are derived from induced mutagenesis rather than natural variation, and their resistance spectra are often narrow, typically conferring protection only against aryloxyphenoxypropionates while remaining ineffective against cyclohexanediones. Likewise, mutations at positions W548M and G628W in the *OsALS* gene confer resistance to ALS-inhibiting herbicides, including imidazolinones [68,69]. Although these ALS mutations provide strong resistance, they often carry a yield penalty under herbicide-free conditions, as the altered ALS enzyme exhibits reduced catalytic efficiency for branched-chain amino acid biosynthesis. Mutations such as T173I and A174V in the *OsEPSPS* gene, as well as target gene amplification, enhance resistance to glyphosate in rice [70,71]. A critical unresolved question is whether EPSPS overexpression imposes a metabolic burden on rice plants, and whether the resistance level conferred by these single amino-acid substitutions is sufficient to withstand field-rate glyphosate applications across different environments. In addition, knockout of the *OsDNR1* gene improves rice tolerance to HPPD-inhibiting herbicides by regulating homogentisate accumulation (Table 3) [72]. While this approach offers a novel NTSR mechanism, its effectiveness is currently limited to HPPD inhibitors, and the potential pleiotropic effects on tyrosine metabolism and plant development remain to be fully evaluated. Overall, resistance mutations identified in rice are mainly concentrated in specific functional domains of the target gene coding regions. This suggests that the spatial conformation of target proteins may be a key factor influencing resistance evolution.

In NTSR studies, the rice endogenous UDP-glycosyltransferase gene *GRGT1* has been shown to reduce glyphosate residues by catalyzing glyphosate glycosylation, thereby enhancing plant tolerance [73]. Despite its novelty, the glycosylation efficiency is substrate-specific and the resistance levels achieved are moderate, making this gene more suitable as a component of gene pyramids rather than a stand-alone resistance trait. The ABC transporter gene *OsABCC8* reduces intracellular glyphosate concentration by effluxing the herbicide from the cytoplasm to the apoplast [66]. An important limitation is whether such transporter-mediated resistance imposes fitness penalties under normal growth conditions due to the energy cost of active efflux, and whether its substrate specificity may limit efficacy against herbicide mixtures. Compared with TSR, NTSR genes generally have broader-spectrum potential but confer weaker resistance effects. This suggests that the breeding application of NTSR may rely more on pyramiding multiple genes or combining with target-site mutations.

Rice possesses the most abundant target-site resistance-gene resources among the four cereals, with multiple validated mutations in *OsALS*, *OsEPSPS*, and *OsACCase*. However, the breeding utilization of endogenous non-target-site resistance genes (e.g., *GRGT1*, *OsABCC8*) remains constrained by their relatively weak resistance levels when expressed alone. Therefore, future rice breeding for herbicide resistance will likely depend on gene pyramiding strategies that combine strong TSR alleles with complementary NTSR modules to achieve field-level durability.

### 5.2. Progress on Herbicide Resistance Genes in Maize

Maize is a major food and feed crop worldwide. Herbicides are indispensable in its large-scale production, but phytotoxicity and herbicide-resistant weeds are also major concerns. In recent years, significant progress has been made in genetic improvement of herbicide resistance in maize, mainly focusing on the discovery and application of resistance genes for glyphosate, glufosinate, and nicosulfuron.

Early efforts to introduce and deploy target-site resistance genes focused largely on transgenic approaches. Glyphosate resistance in maize is primarily achieved through the introduction and stable expression of the *EPSPS* gene, which encodes a glyphosate-insensitive EPSPS protein with low affinity for glyphosate, allowing the enzyme to maintain normal aromatic amino acid synthesis in the presence of the herbicide [74,75]. The major limitation of this approach is the heavy reliance on a single target and a single resistance mechanism, which accelerates the evolution of resistance in weed populations and reduces the durability of the transgenic trait. Glufosinate resistance, on the other hand, relies mainly on the pat gene, which encodes phosphinothricin acetyltransferase that inactivates glufosinate through acetylation. This gene has been successfully deployed in commercial transgenic maize varieties, often stacked with *Bt* insect resistance genes and other herbicide resistance genes for trait stacking [76]. Although gene stacking expands the resistance spectrum, it also raises regulatory complexity and increases the cost of product development. These studies represent the first-generation approach to herbicide resistance breeding in maize—exogenous gene introduction. However, public acceptance of transgenic crops remains a constraint on its further development.

In recent years, the research focus on endogenous resistance genes has gradually shifted from exogenous gene introduction to targeted modification of endogenous genes. For ALS-inhibiting herbicides such as nicosulfuron, Zhang et al. identified *CYP81A9*, encoding a cytochrome P450 monooxygenase, as a key gene regulating nicosulfuron resistance in maize. Its expression level is positively correlated with the activity of the target enzyme ALS. Overexpression of this gene enhances herbicide resistance in maize, whereas gene silencing or loss-of-function mutations leads to loss of resistance (Table 3) [77]. This finding reveals a P450-mediated metabolic detoxification mechanism in maize. Unlike target-site mutations, P450 enzymes reduce the effective herbicide concentration at the target site by accelerating oxidative metabolism, which is a form of NTSR. Nevertheless, the practical application of CYP81A9 faces significant hurdles: its expression is genotype-dependent, its substrate specificity restricts the range of herbicides it can detoxify, and overexpression may impose a metabolic load that affects yield under stress conditions. In addition, the roles of metabolic enzyme genes such as GST in the detoxification of multiple herbicides in maize are gradually being elucidated, providing new theoretical insights for understanding resistance differences among maize germplasms [78,79]. However, most GST studies remain at the transcriptomic level, and functional validation through genetic complementation or knockout is still limited, making it difficult to prioritize specific GST genes for breeding. Notably, gene editing technologies have enabled precise modification of endogenous genes in maize, including target genes such as *ALS* and *EPSPS*, as well as metabolic genes like *CYP81A9*. This allows for the creation of herbicide-resistant germplasm without foreign transgenic components. To some extent, this approach avoids regulatory hurdles and public acceptance issues associated with exogenous gene introduction, while also providing valuable germplasm resources for sustainable and efficient maize production. A key unresolved question is whether edited endogenous alleles can achieve resistance levels comparable to those conferred by transgenic constructs, and whether the editing efficiency in recalcitrant maize genotypes can be sufficiently improved to enable broad application.

In summary, maize leads the four crops in the commercialization of transgenic herbicide resistance, exemplified by *CP4-EPSPS* and pat genes. Nevertheless, the translation of endogenous P450-type NTSR genes (such as *CYP81A9*) to field breeding remains to be advanced, as their expression is often genotype-dependent and their metabolic spectra are not yet fully characterized. Precisely editing these endogenous genes via CRISPR/Cas9 offers a promising route to bypass regulatory constraints associated with transgenesis.

### 5.3. Progress on Herbicide Resistance Genes in Wheat

Wheat is the most widely grown cereal crop in the world. Its large-scale, intensive production relies heavily on chemical weed control. However, long-term, repeated use of the same herbicides not only drives weed resistance but also poses phytotoxicity risks to wheat itself. These challenges have made herbicide resistance research a key component of sustainable weed management in wheat fields. Current research on wheat herbicide resistance mainly focuses on two mechanisms: TSR and NTSR.

In TSR, research has focused on the discovery and precise editing of mutations in two key genes: *TaEPSPS* and *TaALS.* The P106S mutation in *TaEPSPS* reduces glyphosate binding affinity to the target enzyme. By precisely editing this site, glyphosate-resistant wheat germplasm has been successfully developed [80]. However, the P106S mutation alone confers only moderate glyphosate tolerance, and field-level resistance may require stacking with additional mutations or NTSR genes to withstand commercial application rates. Mutations in *TaALS*, such as P174S, G631D, and G632S, significantly enhance wheat tolerance to multiple ALS-inhibiting herbicides, including chlorsulfuron and tribenuron-methyl, and have already been used in breeding resistant varieties (Table 3) [81]. Despite these advances, the number of available resistance mutations reported in wheat is still lower than that in rice, and most have only been validated through in vitro enzyme activity assays or greenhouse pot trials, with limited systematic evaluation of resistance stability and yield performance under field conditions. Moreover, the polyploid nature of wheat necessitates editing all homoeologous copies simultaneously, which remains technically challenging.

In NTSR studies, research has mainly focused on two metabolic enzyme families: CYP450 and GSTs. Upregulation of *CYP72A397*, *CYP72A14*, and *GSTDHAR1* significantly enhances wheat tolerance to pyrazosulfuron, while overexpression of the *CYP81Q32-like* gene reduces wheat sensitivity to sulfentrazone [82,83]. A major limitation is that the herbicide substrates of these P450s are often poorly defined, and their overexpression may affect the metabolism of endogenous hormones or other xenobiotics, potentially leading to unintended agronomic consequences.

In addition, Zhao et al. (2024) performed a genome-wide association study (GWAS) and identified major glyphosate-tolerance QTLs, including qGlyT-1A and qGlyT-6A, on chromosomes 1A and 6A of wheat [84]. These findings provide technical support for rapid screening of resistance genes and marker-assisted selection. However, the candidate genes within these QTLs have not yet been finely mapped or functionally validated. The gap between QTL identification and gene cloning reflects the inherent difficulty of working with the large, repetitive wheat genome, and highlights the need for advanced genomics tools such as long-read sequencing and targeted mutagenesis to accelerate functional validation. This contrasts with the situation in rice, where resistance genes have been cloned to the single-gene level. Such a gap reflects the difficulty posed by the large and complex genome of wheat, which substantially hinders efficient cloning of resistance genes.

To conclude, wheat faces unique challenges due to its large, polyploid, and complex genome, which substantially hampers resistance-gene cloning and QTL fine-mapping compared with rice. Although mutations in *TaEPSPS* and *TaALS* have been validated, and GWAS has identified promising QTLs (qGlyT-1A and qGlyT-6A), functional validation at the single-gene level remains scarce. Future efforts should prioritize high-resolution mapping and the deployment of prime editing to overcome genome redundancy.

### 5.4. Progress on Herbicide Resistance Genes in Sorghum

Compared with rice, maize, and wheat, research on herbicide resistance genes in sorghum started relatively late. However, recent studies have made breakthroughs in two main areas: target enzyme mutations and transgenesis.

Sorghum resistance research targeting enzyme mutations has centered on the *ALS* gene. Using EMS mutagenesis, Tang et al. identified mutations such as A93T and S624N. The *Sbals-1* (A93T) mutant exhibited superior enzyme activity and phenotype under high imidazolinone doses and has been deployed in breeding for cereal–legume intercropping [85]. The value of this finding is that sorghum resistance genes were identified for a specific cropping system, not just for germplasm creation. Cereal–legume intercropping is widely used in dryland farming in northern China, but sorghum’s sensitivity to imidazolinones has long restricted herbicide choices. *Sbals-1* now offers a genetic solution to this bottleneck. This also implies that a resistance gene’s breeding value depends on both its resistance level and its fit with the target cropping system. A critical concern, however, is that the A93T mutation may alter ALS catalytic efficiency, potentially causing a growth or yield penalty, although this has not yet been systematically quantified across different sorghum genetic backgrounds and environments.

In transgenic resistance, *bar* (glufosinate) and *CP4-EPSPS* (glyphosate) have been stably expressed in sorghum. Lines co-transformed with *bar*/*mcry1Ab*/*mvip3A* tolerate 4× the field glufosinate rate and also exhibit insect resistance, showing the feasibility of gene stacking (Table 3) [86,87]. However, compared with other crops, transgenic research in sorghum is still in its early stages. The most critical bottleneck is the extremely low transformation efficiency and strong genotype dependence of sorghum, which severely limits the rapid deployment of both transgenic and gene-edited traits. Moreover, the stability of transgene expression across generations and under field conditions has not been extensively evaluated.

In conclusion, sorghum resistance genes, particularly the *SbALS* (A93T) mutation, primarily serve specific cereal–legume intercropping systems, where they provide a genetic solution to the long-standing herbicide-sensitivity bottleneck. However, broad application of both transgenic (*bar*, *CP4-EPSPS*) and mutant-based traits is severely constrained by low transformation efficiency and strong genotype dependence, which remain key technical barriers to be overcome.

### 5.5. Cross-Crop Comparative Synthesis and Perspectives

The four cereal crops examined above exhibit marked differences in resistance-gene resources, translational progress, and application scenarios. Rice offers the richest target-site allele pool, owing to its compact genome and extensive functional studies, yet the gap between laboratory NTSR validation and field utility remains wide. Maize represents the most advanced case in commercial deployment, but regulatory and public-acceptance issues with transgenes are driving a shift toward endogenous gene editing. Wheat lags notably in cloning efficiency because of its allopolyploid genome, which complicates forward genetics and fine-mapping; its path forward lies in precise editing of homoeologs and marker-assisted stacking. Sorghum occupies a specialized niche for intercropping systems, but poor transformation efficiency and genotype dependency severely restrict trait translation.

Across all four crops, a unifying trend is the shift from single-gene to multi-gene pyramiding and from exogenous transgenes to precise editing of endogenous alleles. Common bottlenecks include insufficient systematic mining of resistance resources from wild relatives, poor understanding of NTSR regulatory networks, and strong genotype dependency in transformation. Future efforts should focus on expanding the resistance-gene pool, optimizing delivery systems, and integrating resistance traits with yield and stress tolerance through coordinated breeding pipelines, thereby supporting sustainable herbicide resistance management.

## 6. Challenges

Currently, herbicide resistance genes have become a cornerstone for herbicide-resistant crop breeding and integrated weed management in farmland. However, prominent bottlenecks remain in four key areas, including genetic resources, mechanisms of action, technological applications, and ecological safety, which constrain their efficient, safe, and sustainable utilization.

Major challenges in genetic resources include limited availability, structural homogeneity, and inefficient discovery and validation systems. Currently, scaled resistance genes are narrowly concentrated in a few targets *(EPSPS*, *ALS*, *ACCase*), with narrow sources and convergent types, making them prone to failure under prolonged use. Discovery of novel, high-level, broad-spectrum genes from wild relatives remains low, and most candidates undergo only preliminary characterization, lacking comprehensive validation across multiple crops, environments, and herbicides, which limits practical application. NTSR genes involved in detoxification and sequestration are limited in number and efficacy, hindering pyramiding.

Mechanisms of herbicide resistance remain poorly elucidated. Target-site research has largely focused on single point mutations, with limited attention to mutant protein stability, substrate binding, and compatibility with endogenous metabolism, hindering the resistance–agronomic trait link. NTSR involves multiple pathways (e.g., detoxification, sequestration), yet their synergistic interactions, spatiotemporal regulation, and signaling remain unclear. Most studies are confined to single-gene validation, lacking systematic evaluation of additive or negative effects after pyramiding, so resistance levels often fall short of field standards. Moreover, environmental plasticity is understudied, limiting prediction of resistance fluctuations.

Technological bottlenecks include CRISPR/Cas9 inefficiencies such as off-target effects, chimerism, low site-specific integration, and suboptimal performance in methylated or complex genomic regions. Genotype-dependent transformation severely limits the conversion of elite varieties, hindering rapid gene deployment. Techniques for simultaneous multi-gene editing, tissue-specific expression, and marker-free selection remain underdeveloped, failing to meet breeding safety and efficiency demands. Breeding cycles are long, and stacking herbicide resistance with yield, quality, and stress tolerance remains difficult, slowing field translation. Beyond technology, commercialization faces high registration fees, IP disputes, prolonged approvals, public skepticism, and market volatility, which often constrain more than technical hurdles.

Ecological safety is compromised by incomplete risk frameworks and the spread of resistance traits. Large-scale herbicide-resistant cropping accelerates weed evolution, generating superweeds and reducing gene durability. Pollen-mediated gene flow to wild relatives disperses resistance and causes ecological disturbance. Current assessments are short-term and lab-based, lacking long-term multi-site monitoring. Data on effects on soil microecology, pollinators, and natural enemies remain scarce, and the fate and bioaccumulation of resistance-gene products and metabolites are poorly understood, undermining robust safety evaluation and public confidence.

## 7. Perspectives

Future research should prioritize safety, efficacy, durability, and environmental compatibility in herbicide-resistant crop development. Breeding strategies should shift from single-trait improvement to multi-gene pyramiding, multi-trait synergy, and sustainable utilization. Particular attention should be paid to the following aspects. First, novel, broad-spectrum, and high-level resistance genes should be systematically explored from microorganisms, wild relatives, and crop germplasm resources. Efforts should focus on creating non-target-site and multi-target composite resistance genes with independent intellectual property, thereby enriching the resistance gene pool. Second, the molecular mechanisms underlying herbicide resistance—including target-site mutation, metabolic detoxification, and transport sequestration—should be comprehensively elucidated. Multi-gene regulatory networks and their interactions with environmental adaptation should also be investigated to provide a theoretical foundation for precision breeding. Third, advanced biotechnological tools, including CRISPR-based genome editing, multi-gene site-specific integration, and high-efficiency transformation, should be optimized to overcome genotype dependency. These efforts will facilitate the establishment of marker-free, safe, and efficient resistance improvement platforms, enabling coordinated enhancement of herbicide resistance, yield potential, product quality, and abiotic stress tolerance. Fourth, comprehensive biosafety assessment frameworks and ecological risk monitoring systems should be developed. Long-term studies should evaluate gene flow, weed resistance evolution, environmental fate, and ecological impacts to support sustainable resistance management strategies. Fifth, multi-trait and stacked-resistance varieties should be integrated with science-based herbicide stewardship, including rotation and mixture applications. This will establish an environmentally sustainable management framework that combines genetic improvement with rational herbicide use. Ultimately, these efforts will facilitate the efficient, safe, and sustainable utilization of herbicide-resistant crops, supporting the environmentally sustainable and high-efficiency development of modern agriculture.

## Figures and Tables

**Table 1:** The classification of Photosynthetic inhibitors.

Classification	Target Site	Chemical Class	Representative Herbicides
Photosystem II inhibitors	D1 protein (Ser 264 or other non-His 215 binders)	Triazines	Ametryn, Atrazine, Prometryn
		Ureas	Chlortoluron, Diuron, Isoproturon
		Triazinones	Hexazinone, Metamitron, Metribuzin
		Uracils	Bromacil
		Phenylcarbamates	Desmedipham, Phenmedipham
		Amides	Propanil
		Triazolones	Amicarbazone
	D1 protein (His 215 binders)	Nitriles	Bromoxynil, Ioxynil octanoate, Bromoxynil octanoate
		Benzothiadiazinones	Bentazone
Photosystem I inhibitors	Electron transfer	Pyridinium	Diquat, Diquat dichloride
Pigment synthesis inhibitors	Phytoene dehydrogenase	phenyl ethers	Diflufenican, Picolinafen
		N-phenyl heterocycles	Flurochloridone
		Diphenyl heterocycles	Fluridone, Flurtamone
	Deoxy-D-xylulose phosphate synthase	Isoxazolidinone	Clomazone, Bixlozone
	4-hydroxyphenylpyruvate dioxygenase	Triketones	benzobicyclon, Mesotrione, Sulcotrione
		Pyrazolones	Topramezone, Cypyrafluone, Fenpyrafluone
		Isoxazolones	Isoxaflutole
		Oxazoles	Flusulfamide, Pyrasulfotole
	Solanesyl diphosphate synthase	Isoxazole	Pyrimisulfan
	Homogentisate solanesyltransferase	/	/
	lycopene cyclase	/	/
	Protoporphyrinogen oxidase	N-phenyl-phthalimides	Flumioxazin, Fluthiacet-methyl, Pentoxazone
		Diphenyl ethers	Acifluorfen, Fomesafen, lactofen
		N-phenyl-triazolinones	Carfentrazone-ethyl, Sulfentrazone
		N-phenyl-oxadiazolones	Oxadiazon, Oxadiargyl
		Phenylpyrazoles	Pyraflufen-ethyl
		Pyrazolopyrazoles	Pyraclonil

**Table 2:** The classification of Biosynthesis inhibitors.

Classification	Target Site	Chemical Class	Representative Herbicides
Amino acid synthesis inhibitors	Acetolactate synthase	Imidazolinones	Imazamox, Imazapic, Imazapyr
		Triazolopyrimidines Group 1	Cloransulam-methyl, Diclosulam, florasulam
		Triazolopyrimidines Group 2	Penoxsulam, Pyroxsulam
		Triazolinones	Flucarbazone-sodium, Thiencarbazone-methyl
		Sulfonylureas	Pyrazosulfuron-ethyl, Rimsulfuron, Sulfometuron-methyl, Tribenuron-methyl, Flucetosulfuron
		Sulfonanilides	Triafamone
		Pyrimidinylbenzoates	Bispyribac-sodium, Pyriminobac-methyl, Pyriftalid
	5-enolpyruvylshikimate-3-phosphate synthase	Organophosphorus	Glyphosate, Glyphosate ammonium, Glyphosate dimethylamine salt, Glyphosate potassium salt
	Glutamine synthetase	Phosphinic acids	Glufosinate, Glufosinate-P
Fatty acid synthesis inhibitors	Acetyl-CoA carboxylase	Cyclohexanediones	Clethodim, Sethoxydim, Tralkoxydim
		Aryloxyphenoxypropionates	Pinoxaden, Haloxyfop-methyl, Fluazifop-P-butyl, Fenoxaprop-P-ethyl, Quizalofop-P-ethyl
		Phenylpyrazolines	Pinoxaden
		Bicyclooctanetriones	Metproxybicyclone
	Very-long-chain fatty acid elongase	α-chloroacetamides	Alachlor, Butachlor, Metazachlor, Metolachlor, Pretilachlor
		Thiocarbamates	Dimepiperate, Molinate, Thiobencarb, Triallate
		Benzofurans	Ethofumesate
		Isoxazolines	Pyroxasulfone
		α-oxyacetamides	Mefenacet, Flufenacet
		α-thioacetamides	Anilofos
		Sulfonamides	Dimesulfazet
	Acyl-ACP thioesterases	Diphenyl heterocycles	Oxaziclomefone
Microtubule Inhibitors	Microtubule assembly inhibitors	Dinitroanilines	Butralin, Prodiamine, Pendimethalin
		Benzamides	Pronamide
		Pyridines	Dithiopyr, Thiazopyr
	Microtubule disruptors	Tetrahydrofurans	Icafolin-methyl
Auxinic inhibitors	Auxin mimics	Phenoxycarboxylic acids	2,4-D sodium salt, 2,4-D 2-ethylhexyl ester, MCPA dimethylamine salt
		6-chloropicolinic acids	Aminopyralid, Clopyralid, Picloram
		6-arylpicolinic acids	Florpyrauxifen, Halauxifen
		Quinolinecarboxylic acids	Quinclorac
		Benzoic acids	Dicamba
		Pyridyloxycarboxylic acids	Fluroxypyr, Triclopyr, Fluroxypyr-meptyl
	Auxin transport inhibitors	/	/
Cellulose biosynthesis inhibitors	/	/	/
Dihydropteroate synthase inhibitors	Dihydropteroate synthase	/	/
Dihydroorotate dehydrogenase inhibitors	Dihydroorotate dehydrogenase	/	/

**Table 3:** Progress in Herbicide Resistance Gene Research in Four Major Cereal Crops.

Crop	Gene	Mutation Site	Resistance Type	Target Herbicides	e.g.
Rice	*OsALS*	W548M, G628W	TSR	Imidazolinones	Chen et al., 2021; Wang et al., 2021
	*OsEPSPS*	T173I, A174V	TSR	Glyphosate	Jiang et al., 2022; Achary et al., 2020
	*OsDNR1*	Knockout	NTSR	HPPD inhibitor	Li et al., 2025
	*GRGT1*	/	NTSR	Glyphosate	Yang et al., 2026
	*OsABCC8*	/	NTSR	Glyphosate	Pan et al., 2021
Maize	*CP4-EPSPS*	/	TSR	Glyphosate	Duff et al., 2025
	*Pat^Ex^*	/	NTSR	Glufosinate	Sivamani et al., 2019
	*CYP81A9*	/	NTSR	Nicosulfuron	Zhang et al., 2024
	*GSTs*	/	NTSR	Metolachlor	Li et al., 2017
Wheat	*TaEPSPS*	P106S	TSR	Glyphosate	Chen et al., 2025
	*TaALS*	P174S, G631D, G632S	TSR	Chlorsulfuron/Tribenuron-methyl	Kumar et al., 2025
	*CYP72A397/CYP72A14/GSTDHAR1*	/	NTSR	Pyrazosulfuron	Yang et al., 2025
	*CYP81Q32-like*	/	NTSR	Sulfentrazone	Sudhakar et al. 2026
Sorghum	*SbALS*	A93T	TSR	Imidazolinone	Tang et al., 2025
	*Bar^Ex^*	/	NTSR	Glufosinate	Liu et al., 2026
	*CP4-EPSPS*	/	TSR	Glyphosate	Xie, 2012

## Data Availability

The original contributions presented in this study are included in the article. Further inquiries can be directed to the corresponding authors.
